# Scalable mechanochemical synthesis of a cyclic dehydroalanine peptide

**DOI:** 10.1039/d6ob00662k

**Published:** 2026-05-08

**Authors:** Jamie C. Thuan, Katelyn M. Barron, Jun Ohata

**Affiliations:** a Department of Chemistry, North Carolina State University Raleigh North Carolina 27695 USA johata@ncsu.edu

## Abstract

Dehydroalanine derivatives are valuable building blocks in organic and biomolecular chemistry fields, and their scalable synthesis represents unmet needs. This work examined previously reported *N*-to-*O* acyl transfer-based condensation reactions of a diketopiperazine derivative (also known as cyclic glycine dimer or glycine anhydride) through a series of reaction optimization processes to identify scalable conditions. Liquid-assisted mechanochemistry proved important for the promotion of the overall efficiency and reproducibility of the condensation reaction between acetyl-diketopiperazine and paraformaldehyde.

## Abstract 2

This work identified liquid-assisted mechanochemistry as a more reliable way to synthesize the dehydroalanine-based diketopiperazine derivative.

## Graphical abstract

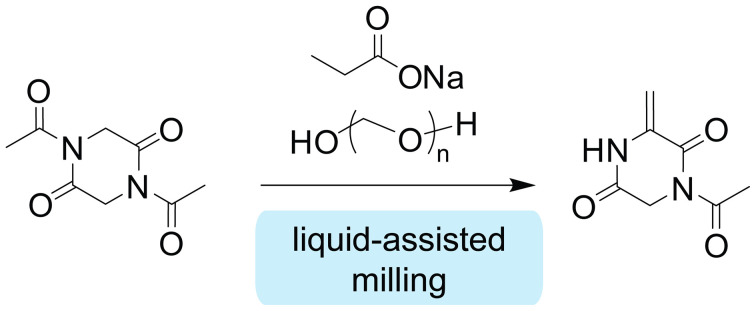

## Introduction

**Fig. 1:**
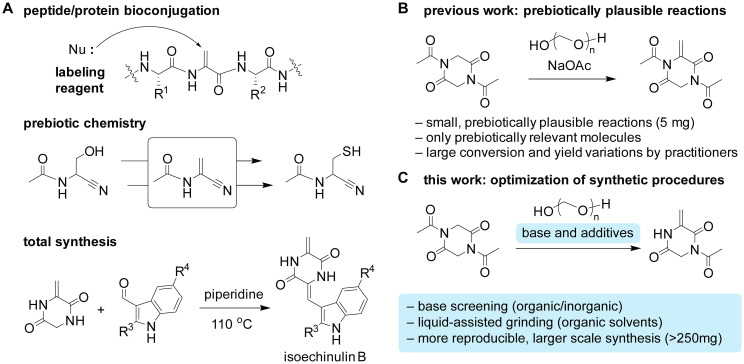
Importance of dehydroalanine-containing compounds. (A) Representative examples highlighting the importance of dehydroalanine derivatives in bioconjugation, prebiotic chemistry, and total synthesis. (B) Previous work focused on the formation of dehydroalanine under prebiotically relevant, small-scale reaction conditions. (C) This work aimed at increasing the reaction scale and reproducibility for preparative synthesis purposes through mechanochemistry using a ball mill.

With the unique reactivity as an electrophile and a radical acceptor in stark contrast to typical natural amino acid residues, dehydroalanine has garnered considerable interest in various organic and biomolecular chemistry fields. Because of the nucleophilic nature of side chains of canonical amino acids and even non-canonical yet natural amino acids (*e.g.*, post-translationally modified amino acids), studies or applications of those amino acids are often enabled by electrophilic reagents and reactants.^1,2^ On the other hand, dehydroalanine bearing an α,β-unsaturated carbonyl group is a unique electrophilic and radical-acceptor amino acid and has been extensively studied during the past years including in the bioconjugation of polypeptides,^3–5^ prebiotically relevant reactions,^6,7^ and as a key intermediate for total synthesis (Fig. 1A).^8^

Dehydroalanine formation can be facilitated by a condensation reaction through an acyl transfer mechanism in a diketopiperazine scaffold, but the efficiency and reproducibility of reactions can vary for formaldehyde substrates. We recently demonstrated the formation of dehydroalanine through an *N*-to-*O* acyl transfer-based condensation reaction between *N*-acetyl-diketopiperazine (*i.e.*, acetylated cyclic glycine dimer or acetylated glycine anhydride) and formaldehyde under prebiotically relevant mechanochemical conditions (Fig. 1B).^9^ The reaction proved more efficient through mechanochemistry over the solution-based ones, although the mechanochemical reaction was tested only at small scales and suffered from reproducibility of reaction yields depending on practitioners. Since the dehydroalanine-containing diketopiperazine can be a useful building block beyond prebiotic chemistry research, this work is focused on optimization and scale increase of the mechanochemical condensation reaction for the preparative purpose. After screening a series of additives, we discovered that liquid-assisted grinding is particularly more effective in increasing the reproducibility and led to a larger scale preparation of the dehydroalanine-containing cyclic peptide. In addition to the unique optimization strategies of the present work compared to a previous report,^10^ utilization of mechanochemical reactions would be important in the peptide science field, as there have not been many reports on peptide substrate examples to date.^11,12^

## Results and discussion

**Fig. 2:**
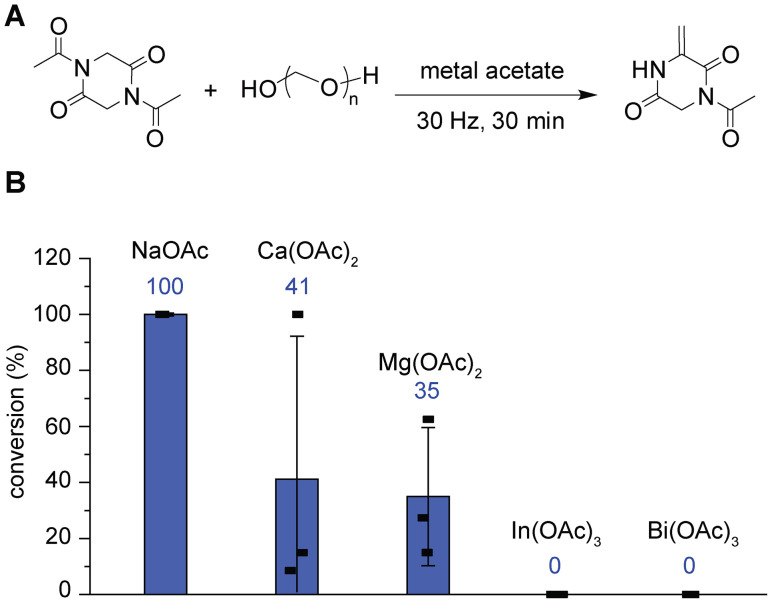
Attempts to identify efficient and reproducible reaction conditions through screening of acetate base additives. Reaction conditions: acetyl diketopiperazine (50 mg, 0.25 mmol), metal acetate (0.85 mmol), and paraformaldehyde (150 mg, 5 mmol per monomeric formaldehyde) at 30 Hz for 30 min using a ball mill. (A) General reaction scheme. (B) Bar graph of NMR analysis of reaction mixtures with metal acetate. OAc = acetate. Salts examined include NaOAc, Ca(OAc)_2_, Mg(OAc)_2_, In(OAc)_3_, and Bi(OAc)_3_. Error bars represent standard deviation (*n* = 3), as more than one practitioner performed experiments to obtain the replicates. The conversion of each replicate is shown as a black rectangle in the bar graph. The averages of the conversions of the replicates are shown above each bar in dark blue. Spectra and NMR yields for the reactions analyzed with an internal standard (1,3,5-trimethoxybenzene) are available in the SI.

The screening of metal acetate salts with varying counter cations revealed that reaction efficiency was associated with the cation type (Fig. 2). In our previous study,^9^ sodium acetate was one of the best bases identified in terms of reaction conversions but with limitations of reproducibility. Therefore, we first examined a series of acetate salts by changing the counter cations to evaluate their effects on the reaction outcome. Sodium, calcium, magnesium, indium, and bismuth acetate salts were chosen for the study, and the reaction mixtures were analyzed by ^1^H NMR. NMR analysis of the calcium and magnesium salt reactions showed substantially less conversion than that of the sodium salt, and the indium and bismuth salts provided no detectable conversions (Fig. 2B, S1 and S2). The lack of reactions may be due to the reported trends in metal–acetate binding strengths,^13^ where interactions between acetate and a higher valent cation can reduce the availability of free acetate relative to sodium acetate (Na^+^ < Ca^2+^ < Mg^2+^ ≪ Bi^3+^ ≈ In^3+^). Although there may be a possibility of slightly increasing the reproducibility by further screening cation types, we decided to change other components to cause a substantial improvement in reproducibility. Additional control experiments indicated that the observed product peaks did not seem to result from the lasting reaction after milling until the NMR sample preparation (aging phenomenon)^14^ as well as from the reaction in a deuterated solvent for the NMR analysis (Fig. S3 and S4). NMR analysis after milling at different time points may also be indicative of the necessity of mechanical inputs for this reaction (Fig. S5).

**Fig. 3:**
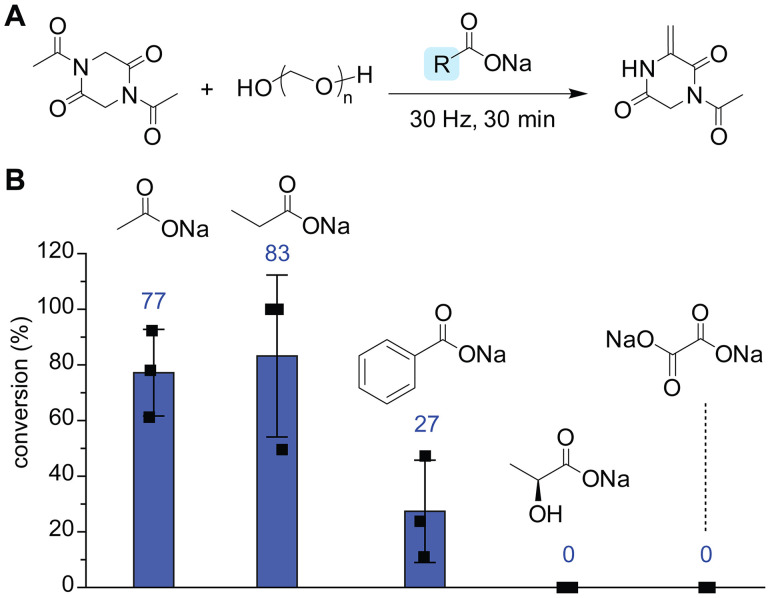
Attempts to identify efficient and reproducible reaction conditions through screening of carboxylate additives. Reaction conditions: acetyl diketopiperazine (50 mg, 0.25 mmol), sodium carboxylate salt (0.85 mmol), and paraformaldehyde (150 mg, 5 mmol per monomeric formaldehyde) at 30 Hz for 30 min using a ball mill. (A) General reaction scheme. (B) Bar graph of NMR analysis of reaction mixtures with carboxylate salts (acetate, propionate, benzoate, lactate, and oxalate). Error bars represent standard deviation (*n* = 3), as more than one practitioner performed experiments to obtain the replicates. The conversion of each replicate is shown as a black rectangle in the bar graph. The averages of the conversions of the replicates are shown above each bar in dark blue. Spectra and NMR yields for the reactions analyzed with an internal standard (1,3,5-trimethoxybenzene) are available in the SI.

Analogous to the effects of cations, screening of types of carboxylate additives did not result in substantial improvement of reaction efficiency and reproducibility (Fig. 3, S6 and S7). As the acetate salt was one of the best bases in our previous study,^9^ our next working hypothesis was to increase the efficiency and reproducibility through structural and electronic alteration of the carboxylic acid substitution. Several commercially available sodium salts of carboxylate compounds such as propionate, lactate, benzoate, and oxalate were chosen to this end. Mechanochemical condensation with those carboxylate salts showed virtually no meaningful improvement from that with the parent acetate salt. For example, no detectable degree of product formation was observed for lactate and oxalate groups, possibly indicative of negative effects of the presence of alkylalcohol and β-keto groups on the condensation reaction. The conversion substantially dropped with the benzoate salt while the use of *n*-propionate salt resulted in a subtle yet probably not statistically significant increase from the acetate one.

**Fig. 4:**
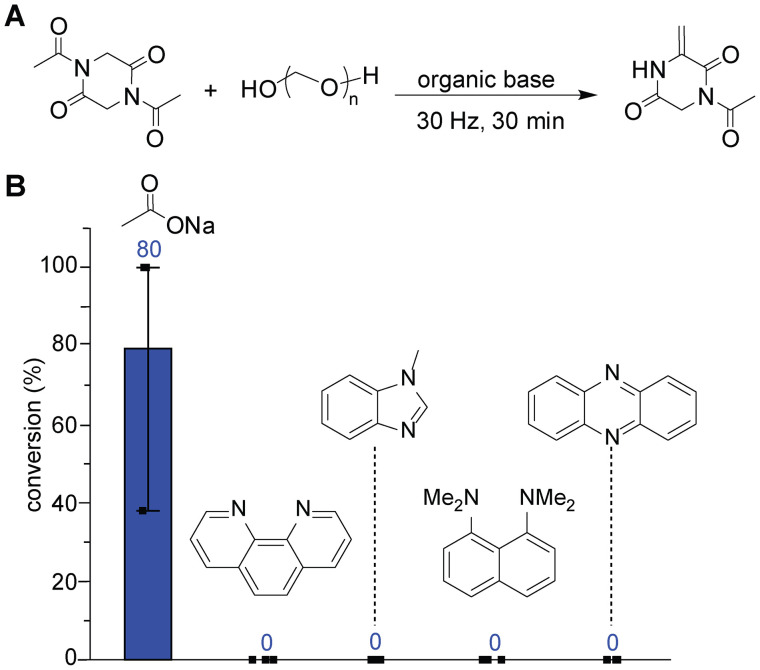
Attempts to identify efficient and reproducible reaction conditions through screening of organic base additives. Reaction conditions: acetyl diketopiperazine (50 mg, 0.25 mmol), organic bases (0.85 mmol), and paraformaldehyde (150 mg, 5 mmol per monomeric formaldehyde) at 30 Hz for 30 min using a ball mill. (A) General reaction scheme. (B) Bar graph of NMR analysis of reaction mixtures with acetate and organic bases (1,10-phenanthroline, *N*-methyl benzimidazole, proton sponge, and phenazine). Error bars represent standard deviation (*n* = 3), as more than one practitioner performed experiments to obtain the replicates. The conversion of each replicate is shown as a black rectangle in the bar graph. The averages of the conversions of the replicates are shown above each bar in dark blue. Spectra and NMR yields for the reactions analyzed with an internal standard (1,3,5-trimethoxybenzene) are available in the SI.

When using organic base additives, there was no appreciable reactivity and the reactions with organic bases did not result in an increase in reaction efficiency or reproducibility. As our previous report demonstrated,^9^ there is tolerance for basicity in this reaction, which provided the motivation to test a range of prebiotically irrelevant organic bases. Organic bases with varied basicity were selected including phenazine (p*K*_a_ 1.2),^15^ phenanthroline (p*K*_a_ 4.9),^16^ methyl benzimidazole (p*K*_a_ 5.6),^17^ and proton sponge (p*K*_a_ 12.1).^18^ The mechanochemical reactions with those organic bases did not show any evidence for product formation based on NMR analysis (Fig. 4, S8, and S9). Because the range of inorganic and organic bases did not offer the chance to increase efficiency and reproducibility dramatically, we turned our attention to a unique reactivity and selectivity alteration method called liquid-assisted grinding.

**Fig. 5:**
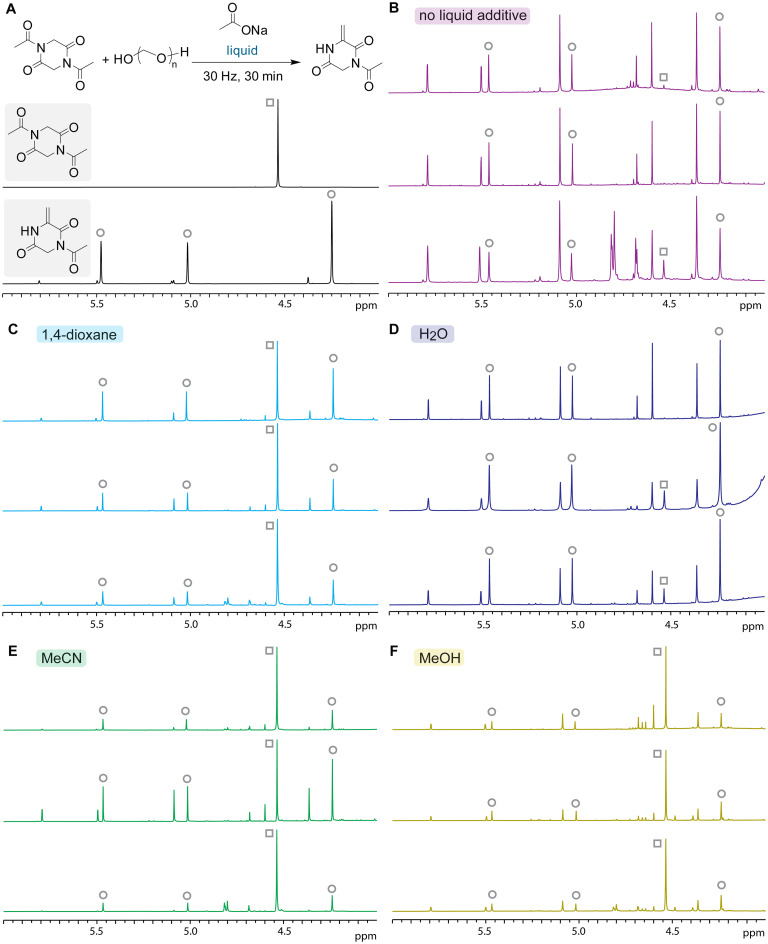
NMR analysis of liquid-assisted grinding to achieve reproducible dehydroalanine formation. Gray circle: peaks of the product. Gray square: a peak of the starting material. Reaction conditions: acetyl DKP (50 mg, 0.25 mmol), sodium acetate (150 mg, 1.82 mmol), and paraformaldehyde (150 mg, 5 mmol per monomeric formaldehyde) liquid (20 μL) at 30 Hz for 30 min using a ball mill. The *η*-value for the liquid-assisted grinding is 0.057 μL mg^−1^.^20^ (A) General reaction scheme and NMR spectra of the starting material and the desired product. (B–F) Partial NMR spectra (4–6 ppm) of reaction mixtures in DMSO-*d*_6_ after milling using no additional liquid (B), 1,4-dioxane (C), water (D), acetonitrile (E), and methanol (F). Three independent experiments by two practitioners are shown for each condition. Spectra and NMR yields for the reactions analyzed with an internal standard (1,3,5-trimethoxybenzene) are available in the SI.

Inclusion of a small amount of a common organic solvent, known as liquid-assisted grinding, showed more reproducible formation of the desired dehydroalanine product in a more modest conversion (Fig. 5). Liquid-assisted mechanochemistry has proved its usefulness in numerous types of chemical reactions for various purposes.^19^ Inspired by such a large body of literature, a set of common solvents such as water, methanol, ether, and acetonitrile was applied to the mechanochemical condensation of acetyl-diketopiperazine (Fig. S10–S15). While most of these solvents caused either virtually no effects or random increase/decrease of reaction efficiency, we identified that the reaction with a small amount of 1,4-dioxane tends to generate the desired product more exclusively without significant generation of unidentified side products (Fig. 5C and S11). To confirm the reproducibility of the result, the same experiments of the liquid-assisted mechanochemistry were repeated three times by two practitioners, and the partial NMR spectra are shown in Fig. 5B–F. For example, milling without liquid additives (Fig. 5B) resulted in substantial consumption of the starting material (peaks labeled with gray square), although peaks of not only the desired product (peaks labeled with gray circle) but also other unidentified peaks such as those at 4.8 and 5.5 ppm were observed in varying degrees in each replicate (Fig. S10). Another example is milling with acetonitrile as shown in Fig. 5E where the amount/ratio of the remaining starting material and desired product, and random peaks after the reaction vary in every experiment (Fig. S13). Because separation of the starting material is more facile than that of unidentified compounds from the desired product during purification, 1,4-dioxane would offer the most reliable and reproducible preparation of the condensation product among the tested liquid-assisted conditions, even if the dioxane-assisted milling would end up in a modest yield due to the relatively low conversion. While it remains unclear why 1,4-dioxane is effective in suppression of the potential side products, it is possible that this reaction proceeds through an *N*-to-*O* acyl transfer mechanism with a carboxylate salt as a base (Fig. S16). In order to make sure that the remaining carboxylate salts are not causing any unwanted side reactions after the milling process, we also compared the reaction outcomes with and without ammonium chloride addition, but the effect of the addition does not seem very significant (Fig. S17). With the dioxane-assisted procedure, we were able to scale up the reaction that produced approximately 250 mg of the desired product using either sodium acetate or sodium propionate salts (procedures available in the SI), and those procedures would be of great value for further applications of dehydroalanine-based DKPs.

## Conclusions

Liquid-assisted mechanochemical aldol condensation has been showed to be important for dehydroalanine formation in a diketopiperazine motif. While this work focused on laboratory-scale reactions, there is potential for the liquid-assisted mechanochemical condensation reaction to be used at an industry level. For example, solid paraformaldehyde is a relatively safer donor than its aqueous solution, and the mechanochemical approach circumvents the common solubility challenges of the solid paraformaldehyde. The need for only a small amount of organic solvent is another attractive feature for industrial applications from the standpoints of safety and environment/waste as well.^21^ Even if our research group's primary interest in dehydroalanine derivatives is centered on prebiotic chemistry, the cyclic dehydroalanine–acetylglycine product would be useful as a building block with nucleophilic and electrophilic reactivities for a broad chemistry community; the α,β-unsaturated carbonyl unit can be used for nucleophilic or radical addition^22^ (or for another reaction such as a Diels–Alder reaction, although stereocontrol could be a challenge)^23,24^ whereas the remaining acetylglycine unit can undergo another condensation reaction with an aldehyde electrophile.^25,26^

## Conflicts of interest

The authors declare no conflict of interest.

## Supplementary Material

OB-024-D6OB00662K-s001

## Data Availability

The data supporting this article have been included as part of the supplementary information (SI). Supplementary information: supplementary figures, general procedure, equipment, synthetic procedure, and the ^1^H NMR spectrum of the final product. See DOI: https://doi.org/10.1039/d6ob00662k.
