# Supplementary material for: Scalable mechanochemical synthesis of a cyclic dehydroalanine peptide
Source: Org Biomol Chem. 2026 May 8;24(20):4242–7. doi: 10.1039/d6ob00662k (PMC13174139; doi:10.1039/d6ob00662k)
Supplement: OB-024-D6OB00662K-s001 [file OB-024-D6OB00662K-s001.pdf]

Supplementary Information (SI) for Organic & Biomolecular Chemistry.
This journal is © The Royal Society of Chemistry 2026

Electronic Supporting Information

Scalable mechanochemical synthesis of a cyclic dehydroalanine peptide

Jamie C. Thuan,‡ Katelyn M. Barron,‡ Jun Ohata*

Department of Chemistry, North Carolina State University, Raleigh, North Carolina 27695, United States.

Email: johata@ncsu.edu

‡: These authors contributed equally to this work.

Table of Contents

Supporting Figures 2

General Information 19

Materials and reagents 19

List of materials 19

Instrumentation 19

Experimental procedures 20

Preparative synthesis of small molecules 20

Organic synthesis procedures 20

NMR spectra of synthesized compounds 22

References for Supporting Information 23

Supporting Figures

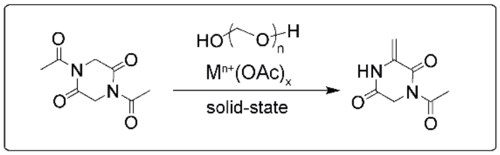

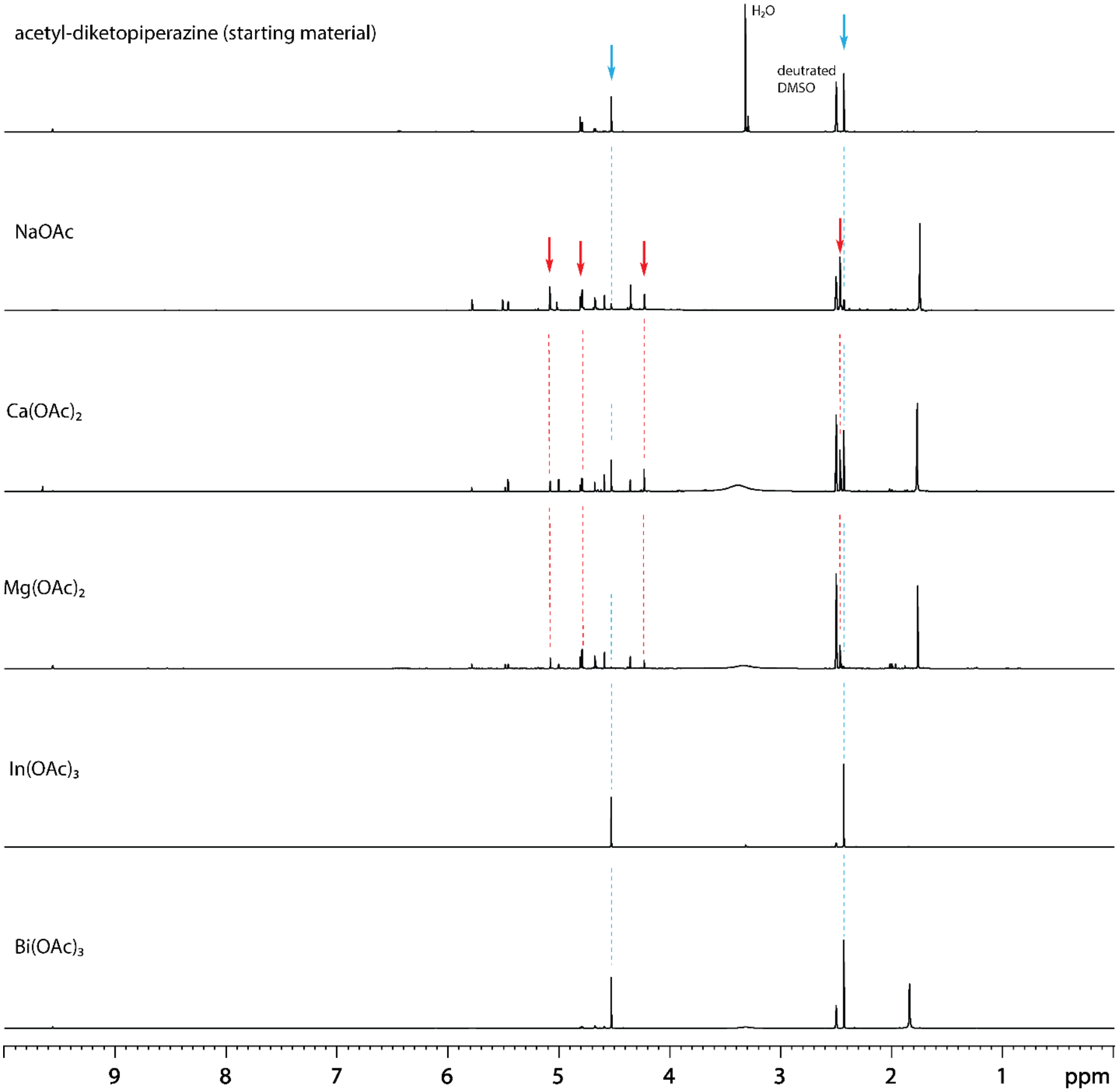

Figure S1. ^1H-NMR spectra (600 MHz, DMSO-d_6) of reaction mixtures after milling with various acetate salts. The starting material acetyl-diketopiperazine and cyclic N-acetyl glycine dehydroalanine are labeled with blue and red arrows (as well as dotted lines), respectively. Reaction conditions: N-acetyl cyclic glycine dimer (50 mg, 1 eq), paraformaldehyde (150 mg, ca. 20 eq), and salts (150 mg) at rt for 30 min in a 1.5 mL milling jar with stainless steel balls (one 3-mm and two 2-mm balls).

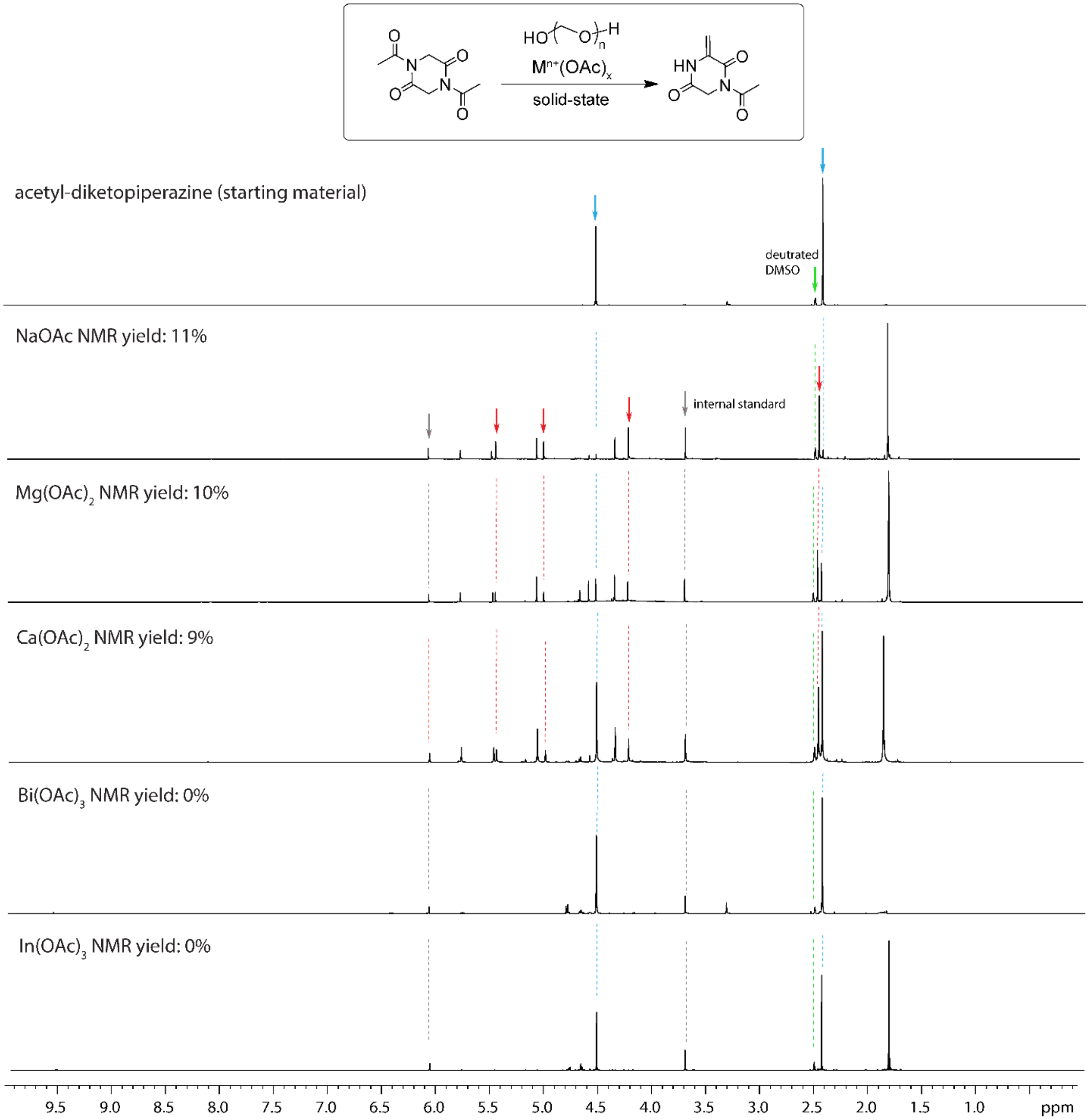

Figure S2. ^{1}H NMR spectra (700 MHz, DMSO-d_6) of reaction mixtures analyzed with 6.7 mM internal standard of 1,3,5-trimethoxybenzene in DMSO-d_6 after milling with various acetate salts. The starting material acetyl-diketopiperazine, cyclic N-acetylglycine dehydroalanine, and internal standard 1,3,5-trimethoxybenzene are labeled with blue, red, and grey arrows (as well as dotted lines), respectively. Reaction conditions: N-acetyl cyclic glycine dimer (50 mg, 1 eq), paraformaldehyde (150 mg, *ca*. 20 eq), and salts (150 mg) at rt for 30 min in a 1.5 mL milling jar with stainless steel balls (one 3-mm and two 2-mm balls).

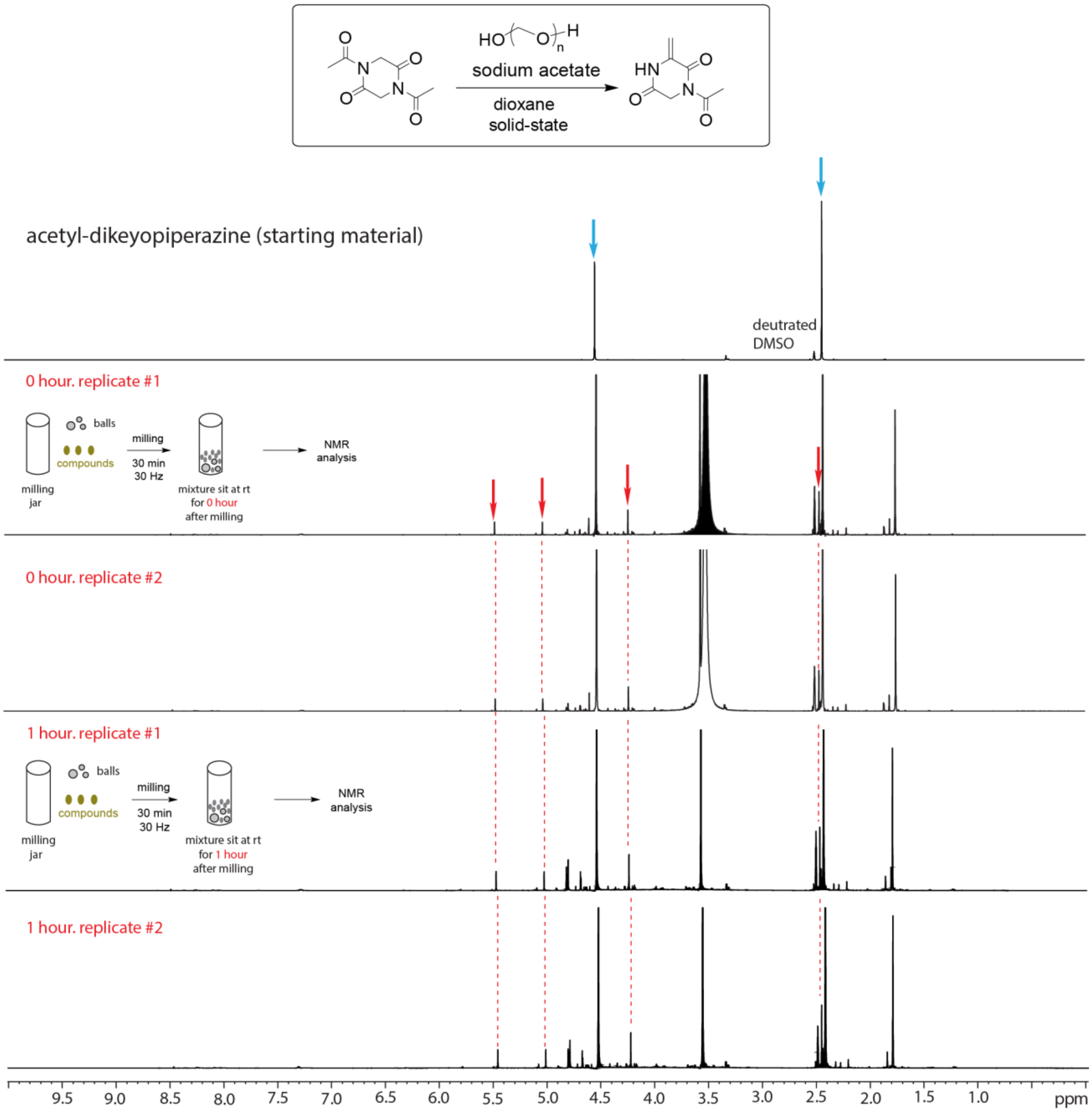

Figure S3.^{1}H NMR spectra (700 MHz, DMSO-d_6) of reaction mixtures analyzed immediately (0 hour) or 1 hour after the milling process. The starting material acetyl-diketopiperazine and cyclic N-acetylglycine dehydroalanine are labeled with blue and red arrows (as well as dotted lines), respectively. Reaction conditions: N-acetyl cyclic glycine dimer (50 mg, 1 eq), paraformaldehyde (150 mg, ca. 20 eq), sodium acetate (150 mg, 7.3 eq), and dioxane (20 uL) at rt for 30 min in a 1.5 mL milling jar with stainless steel (one 3-mm and two 2-mm balls). After milling, the two bottom spectra were allowed to sit for 1 hour before analyzing by ^{1}H NMR.

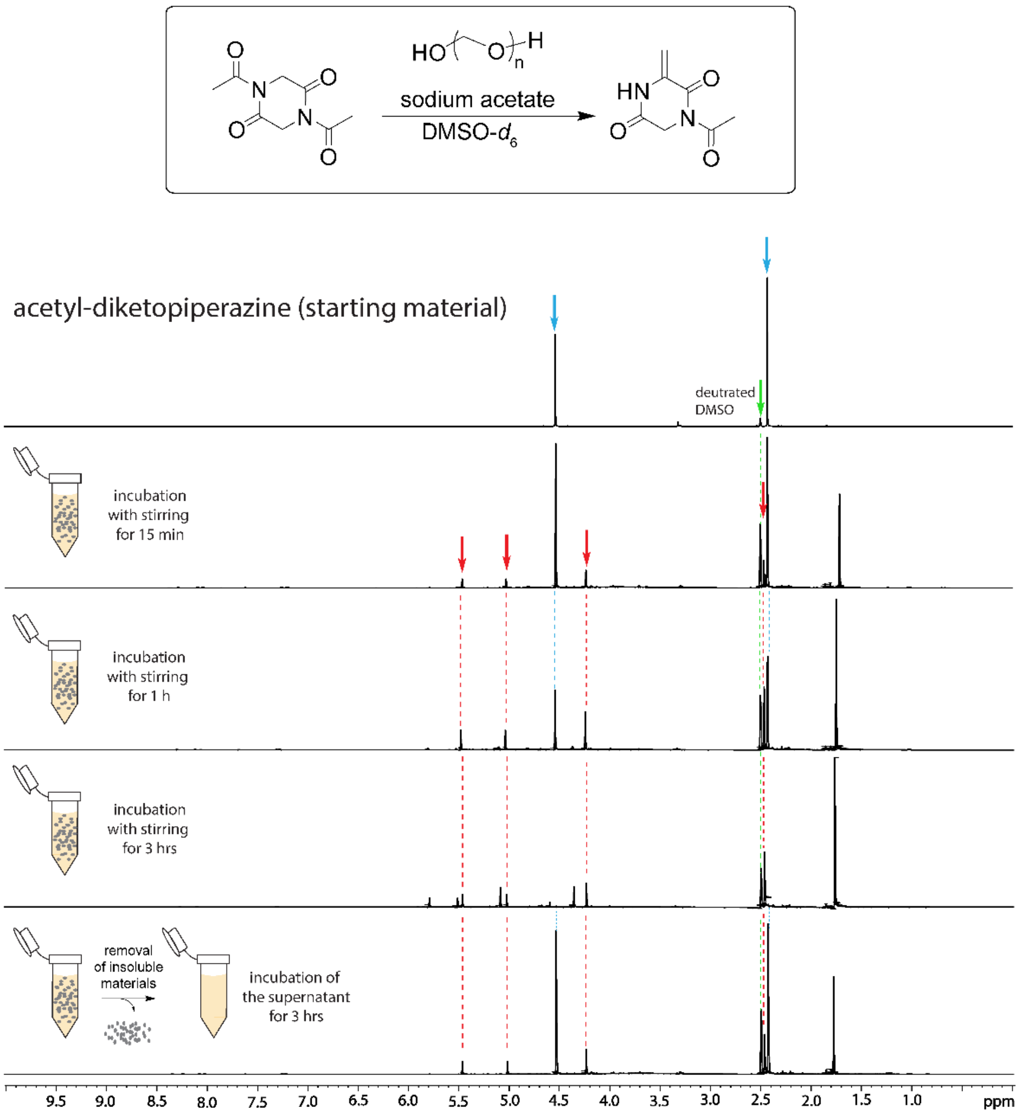

Figure S4.^{1}H NMR spectra (700 MHz, DMSO-d_6) of reaction mixtures analyzed in DMSO-d_6, testing potential reactions of solid mixtures after addition of the deuterated solvent (aging phenomenon). The starting material acetyl-diketopiperazine and cyclic N-acetylglycine dehydroalanine are labeled with blue and red arrows (as well as dotted lines), respectively. Reaction conditions: N-acetyl cyclic glycine dimer (5 mg), paraformaldehyde (5 mg), and sodium acetate (5 mg) in DMSO-d_6 at rt. The reaction of the bottom spectrum was performed after removal of insoluble materials by centrifugation.

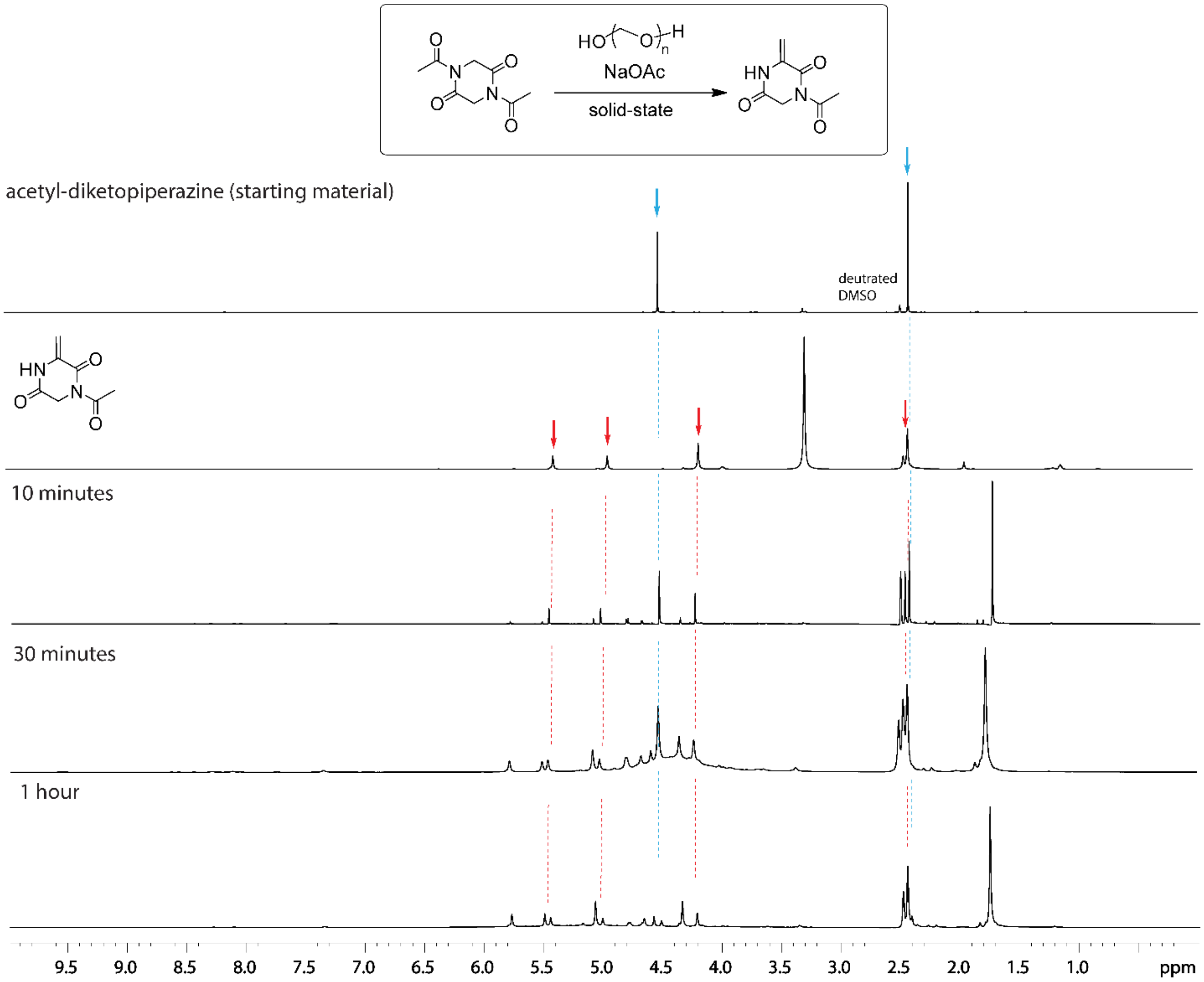

Figure S5.^{1}H NMR spectra (600 MHz, DMSO-d_6) of reaction mixtures analyzed at 10 minutes, 30 minutes, and 1 hour in DMSO-d_6 after milling with sodium acetate. The starting material acetyl-diketopiperazine and cyclic N-acetylglycine dehydroalanine are labeled with blue and red arrows (as well as dotted lines), respectively. Reaction conditions: N-acetyl cyclic glycine dimer (50 mg, 1 eq), paraformaldehyde (50 mg, *ca*. 7 eq), and sodium acetate (50 mg, *ca.* 3 eq) at rt for 30 min in a 1.5 mL milling jar with stainless steel balls (one 3-mm and two 2-mm balls).

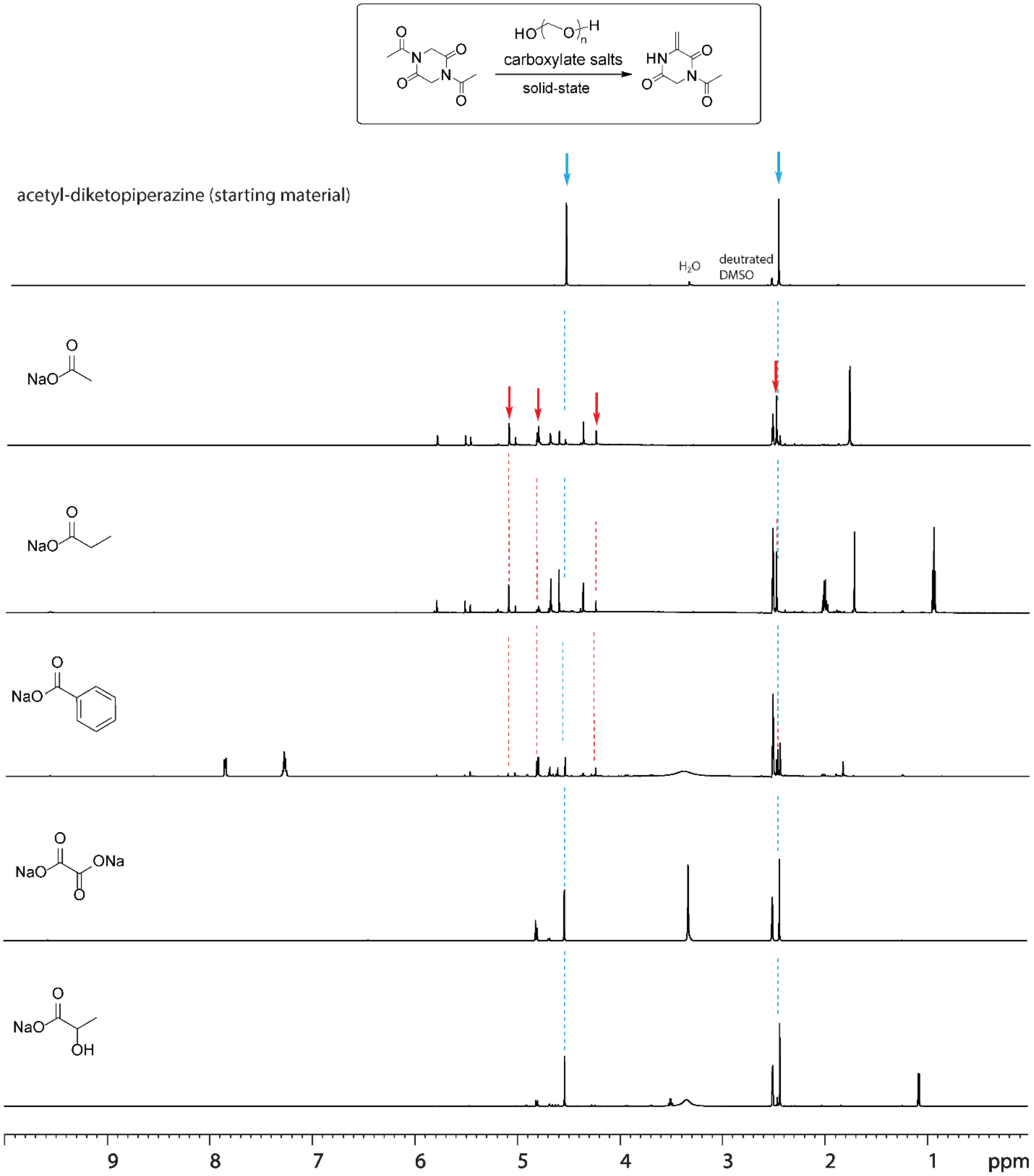

Figure S6 . ^{1}H-NMR spectra (600 MHz, DMSO- d_6) of reaction mixtures after milling with various carboxylate salts. The starting material acetyl-diketopiperazine and cyclic N-acetyl glycine dehydroalanine are labeled with blue and red arrows (as well as dotted lines), respectively. Reaction conditions: N-acetyl cyclic glycine dimer (50 mg, 1 eq), paraformaldehyde (150 mg, ca. 20 eq), and salts (150 mg) at rt for 30 min in a 1.5 mL milling jar with stainless steel balls (one 3-mm and two 2-mm balls).

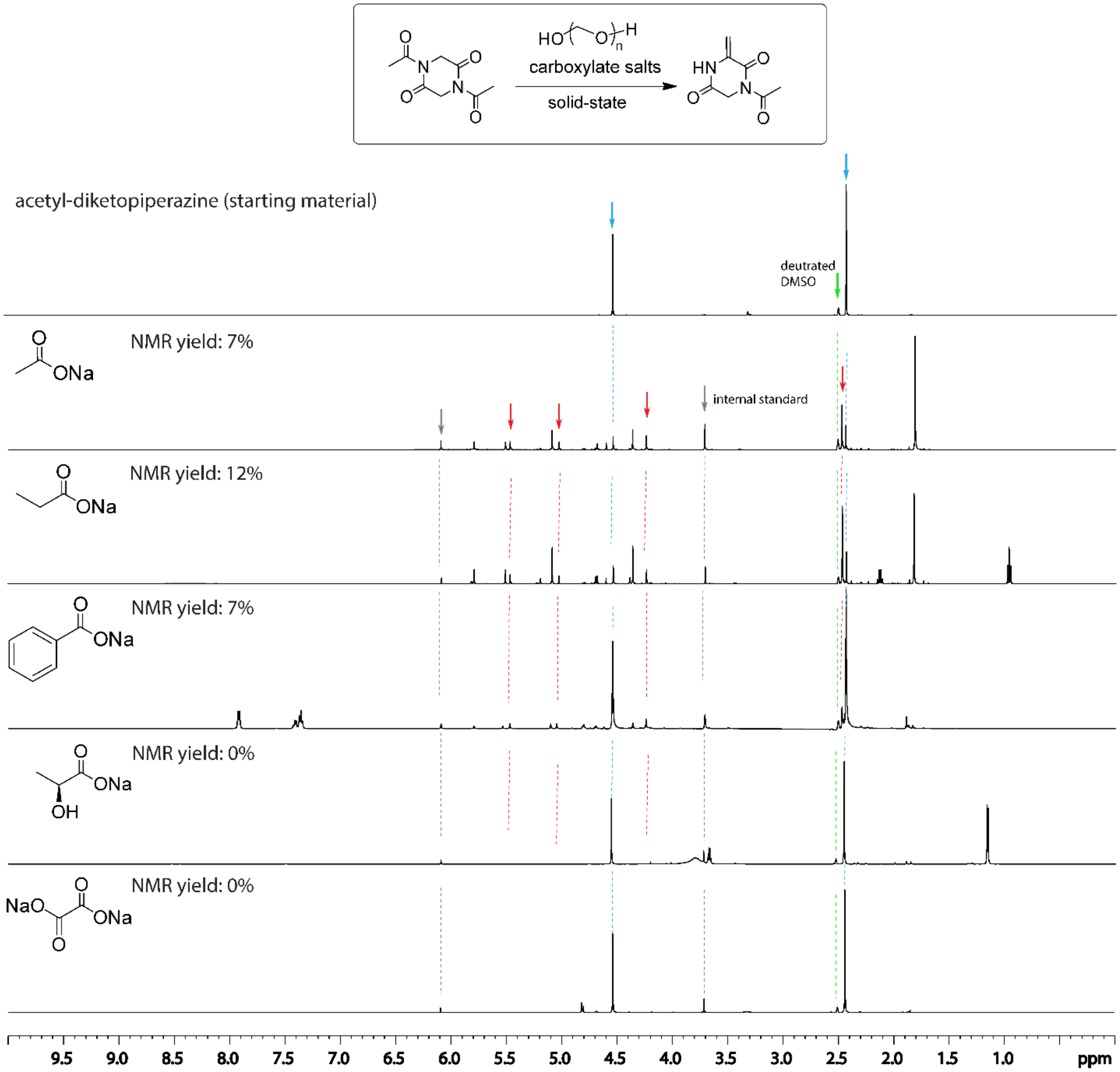

Figure S7. ^{1}H NMR spectra (700 MHz, DMSO-d_6) of reaction mixtures analyzed with 6.7 mM internal standard of 1,3,5-trimethoxybenzene in DMSO-d_6 after milling with various carboxylate salts. The starting material acetyl-diketopiperazine, cyclic N-acetylglycine dehydroalanine, and internal standard 1,3,5-trimethoxybenzene are labeled with blue, red, and grey arrows (as well as dotted lines), respectively. Reaction conditions: N-acetyl cyclic glycine dimer (50 mg, 1 eq), paraformaldehyde (150 mg, *ca*. 20 eq), and salts (150 mg) at rt for 30 min in a 1.5 mL milling jar with stainless steel balls (one 3-mm and two 2-mm balls).

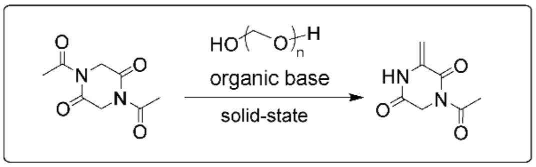

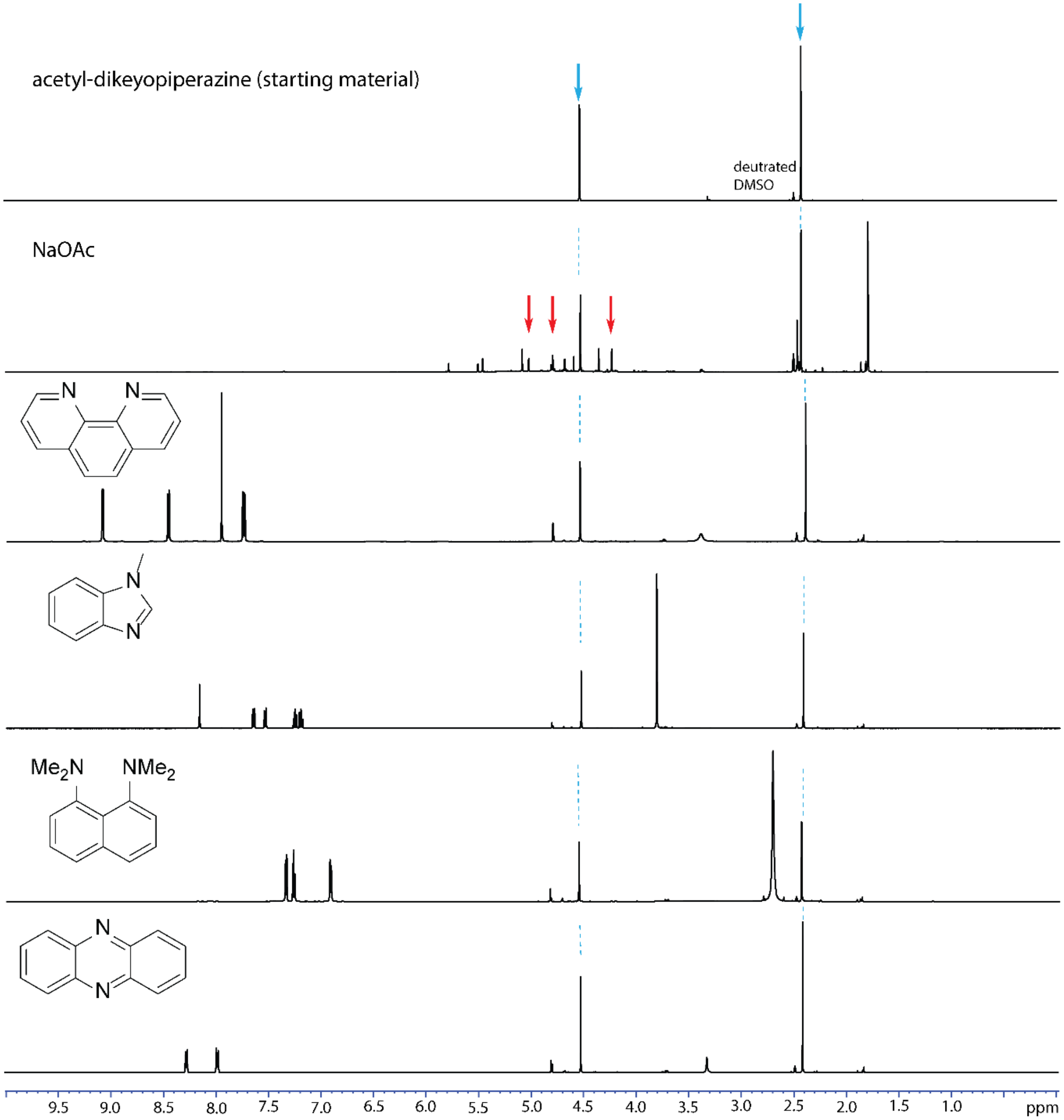

Figure S8. ^{1}H-NMR spectra (600 MHz, DMSO- d_6) of reaction mixtures after milling with various organic bases. The starting material acetyl-diketopiperazine and cyclic N-acetyl glycine dehydroalanine are labeled with blue and red arrows (as well as dotted lines), respectively. Reaction conditions: N-acetyl cyclic glycine dimer (50 mg, 1 eq), paraformaldehyde (150 mg, ca. 20 eq), and organic bases (150 mg) at rt for 30 min in a 1.5 mL milling jar with stainless steel balls (one 3-mm and two 2-mm balls).

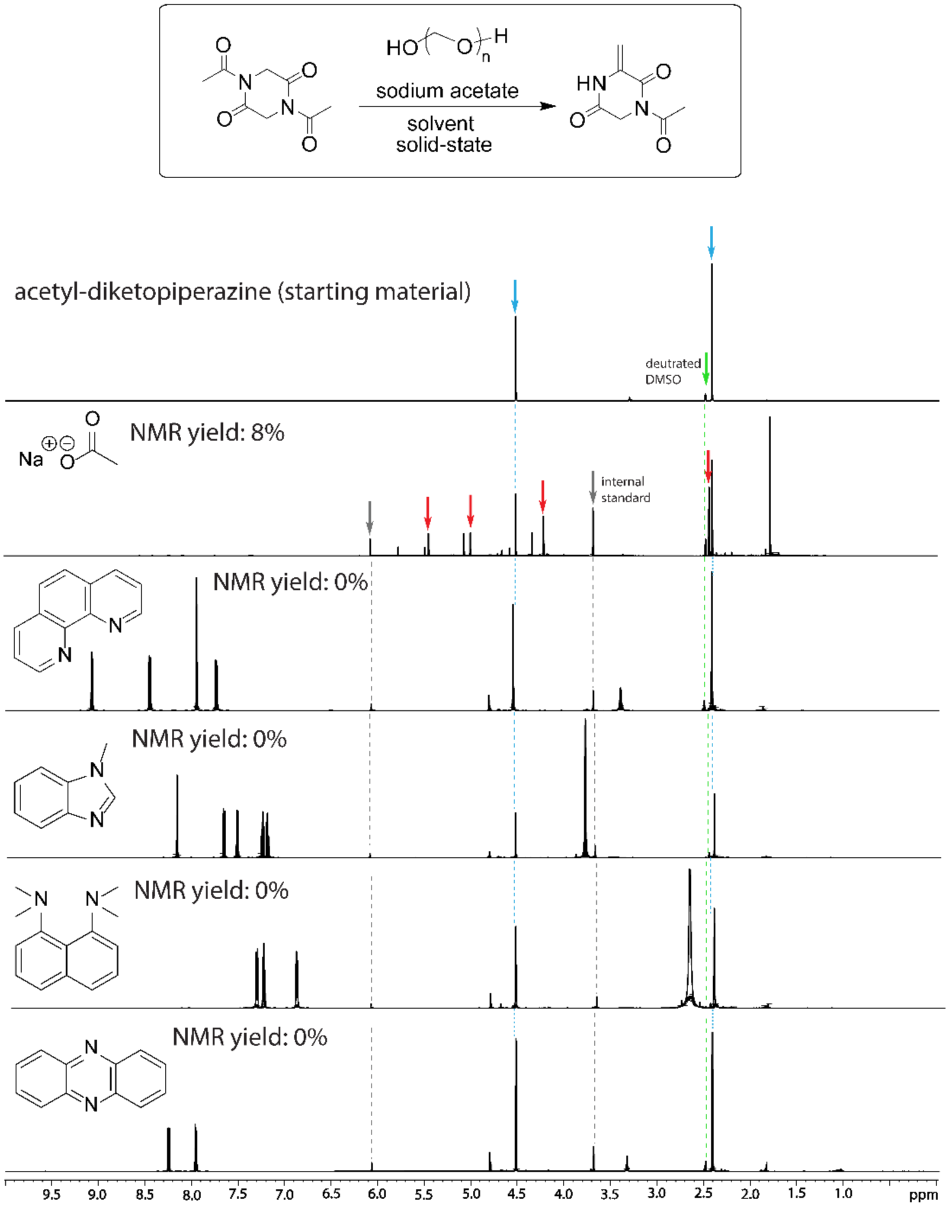

Figure S9. ^{1}H NMR spectra (700 MHz, DMSO-d_6) of reaction mixtures analyzed with 6.7 mM internal standard of 1,3,5-trimethoxybenzene in DMSO-d_6 after milling with various organic bases. The starting material acetyl-diketopiperazine, cyclic N-acetylglycine dehydroalanine, and internal standard 1,3,5-trimethoxybenzene are labeled with blue, red, and grey arrows (as well as dotted lines), respectively. Reaction conditions: N-acetyl cyclic glycine dimer (50 mg, 1 eq), paraformaldehyde (150 mg, *ca*. 20 eq), and organic bases (150 mg) at rt for 30 min in a 1.5 mL milling jar with stainless steel balls (one 3-mm and two 2-mm balls).

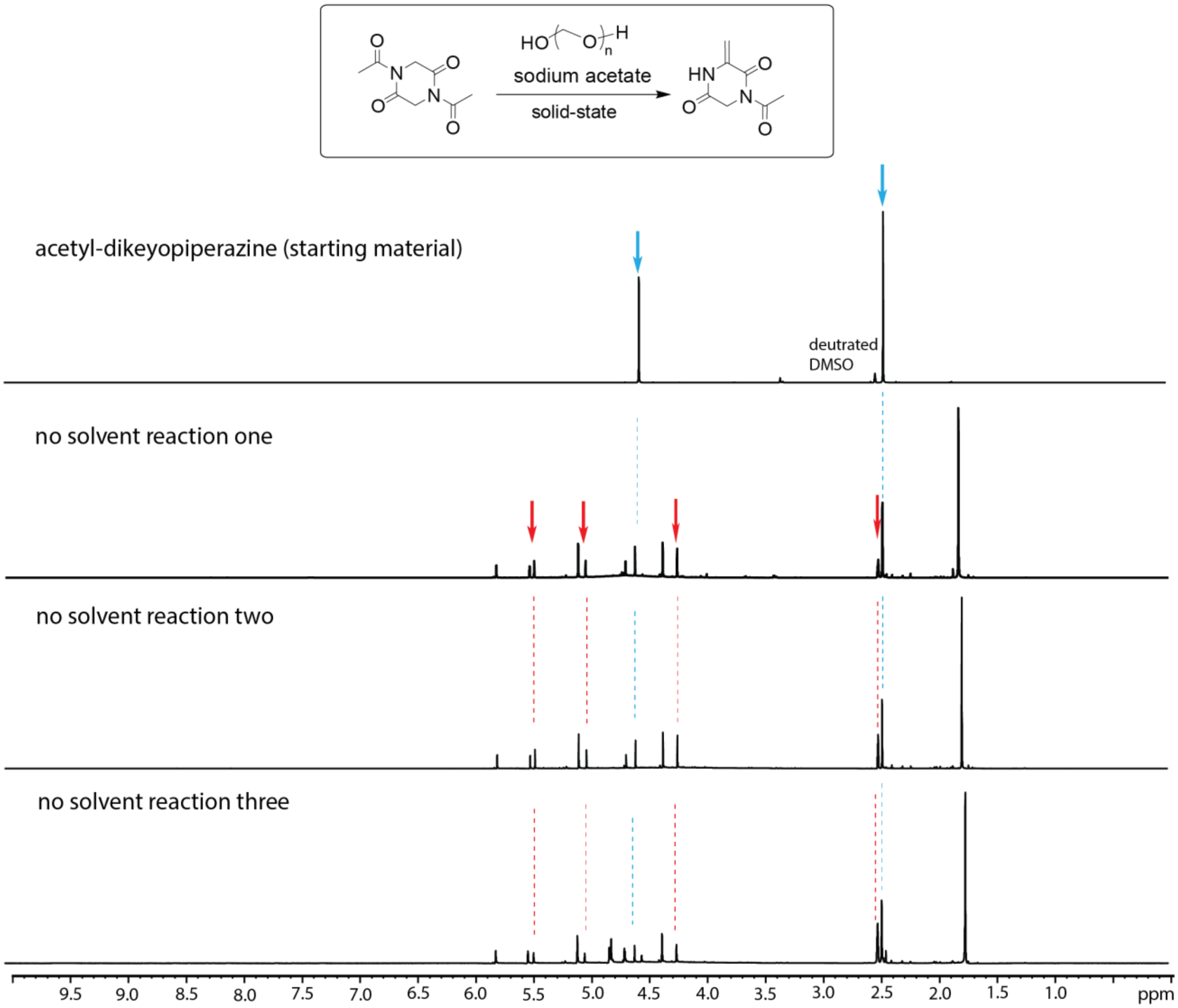

Figure S10 . ^{1}H NMR spectra (600 MHz, DMSO-d6) of reaction mixtures after milling with no liquid additives. The starting material acetyl-diketopiperazine and cyclic N-acetylglycine dehydroalanine are labeled with blue and red arrows (as well as dotted lines), respectively. Reaction conditions: N-acetyl cyclic glycine dimer (50 mg, 1 eq), paraformaldehyde (150 mg, ca. 20 eq), and sodium acetate (150 mg) at rt for 30 min in a 1.5 mL milling jar with stainless steel balls (one 3-mm and two 2-mm balls).

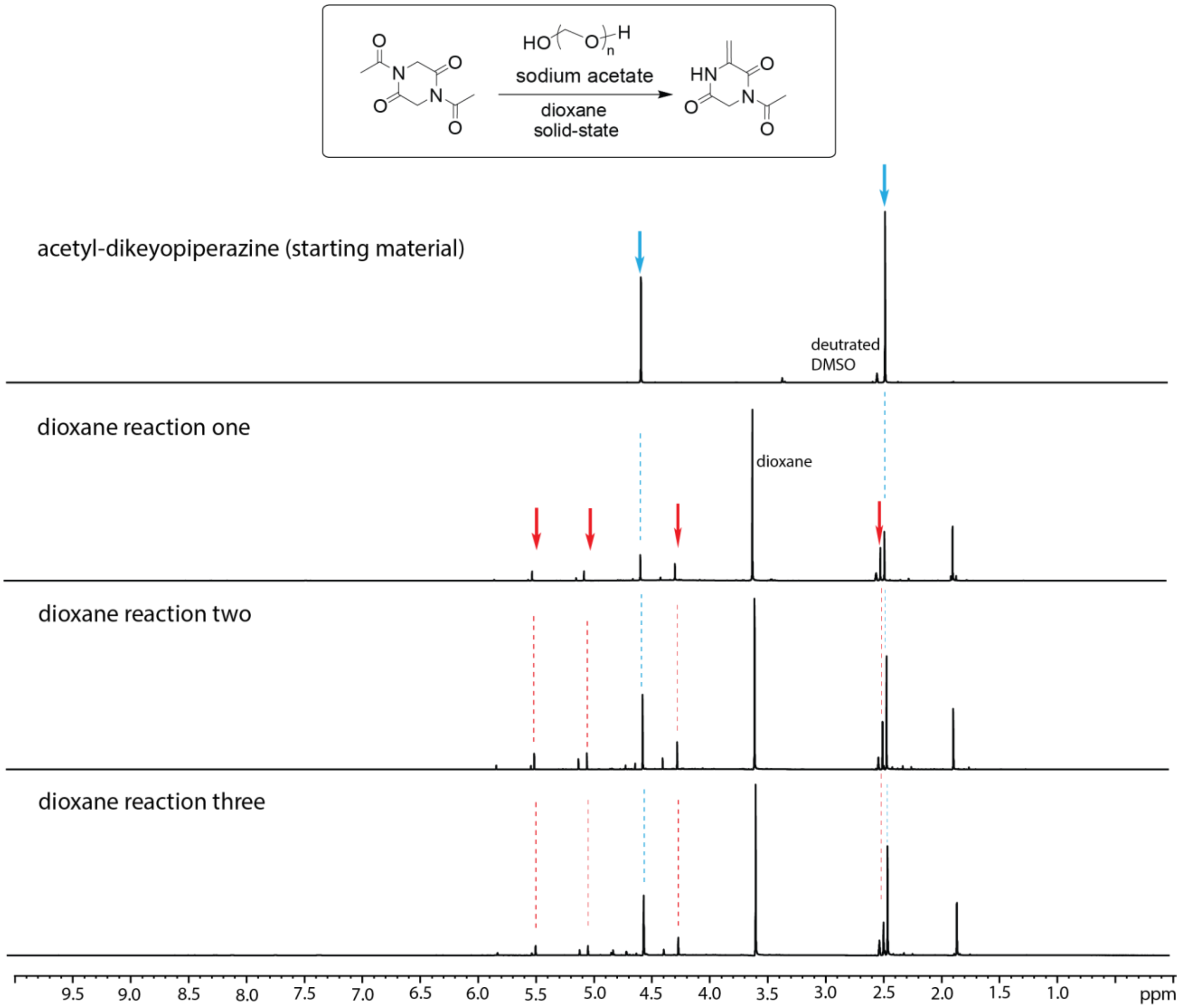

Figure S11. ^{1}H NMR spectra (600 MHz, DMSO-d_6) of reaction mixtures after milling with dioxanes as liquid additive. The starting material acetyl-diketopiperazine and cyclic N-acetylglycine dehydroalanine are labeled with blue and red arrows (as well as dotted lines), respectively. Reaction conditions: N-acetyl cyclic glycine dimer (50 mg, 1 eq), paraformaldehyde (150 mg, ca. 20 eq), sodium acetate (150 mg), and dioxanes (20 µL) at rt for 30 min in a 1.5 mL milling jar with stainless steel balls (one 3-mm and two 2-mm balls).

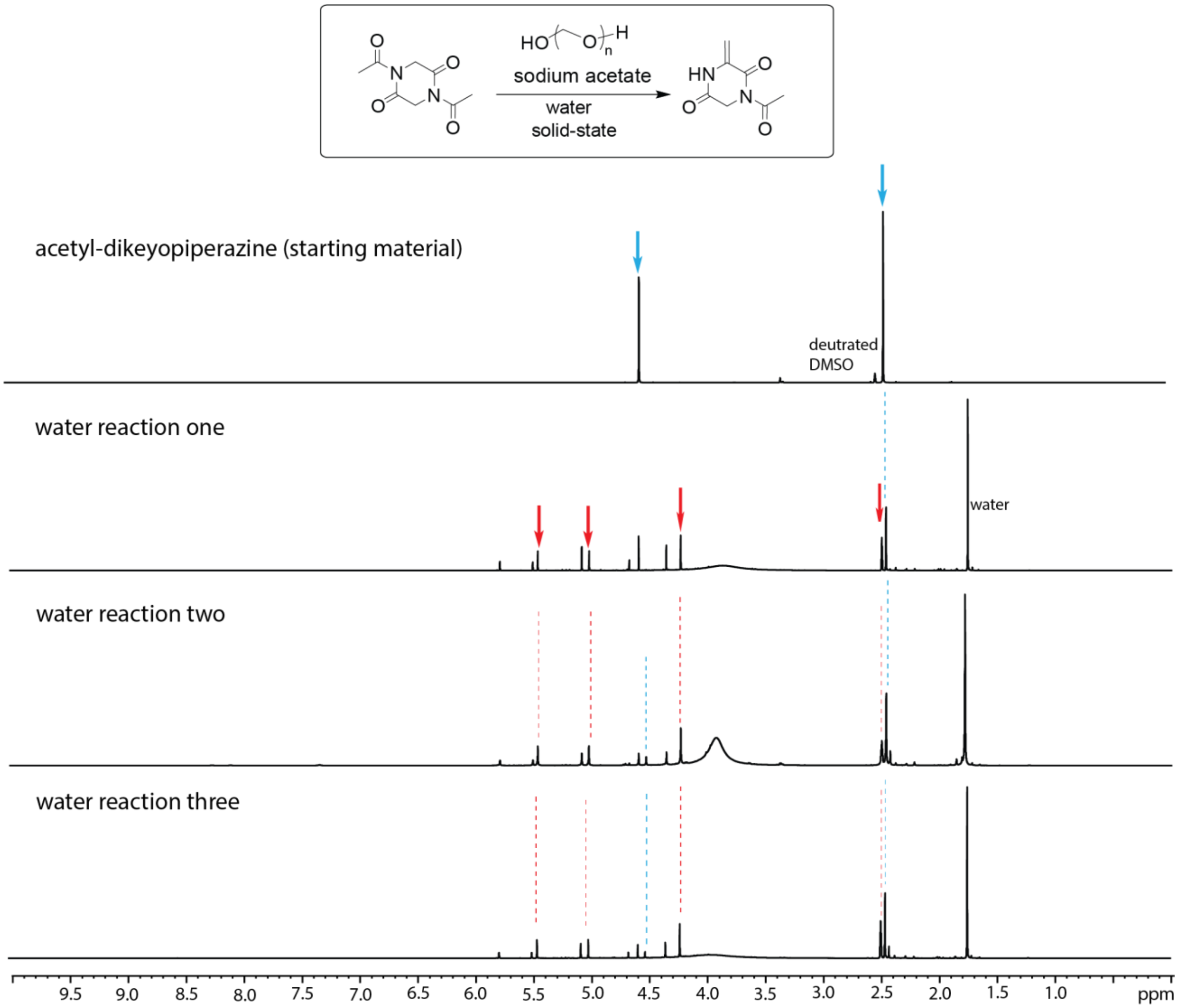

Figure S12 . ^{1}H-NMR spectra (600 MHz, DMSO-d_6) of reaction mixtures after milling with water as liquid additive. The starting material acetyl-diketopiperazine and cyclic N-acetylglycine dehydroalanine are labeled with blue and red arrows (as well as dotted lines), respectively. Reaction conditions: N-acetyl cyclic glycine dimer (50 mg, 1 eq), paraformaldehyde (150 mg, ca. 20 eq), sodium acetate (150 mg), and water (20 µL) at rt for 30 min in a 1.5 mL milling jar with stainless steel balls (one 3-mm and two 2-mm balls).

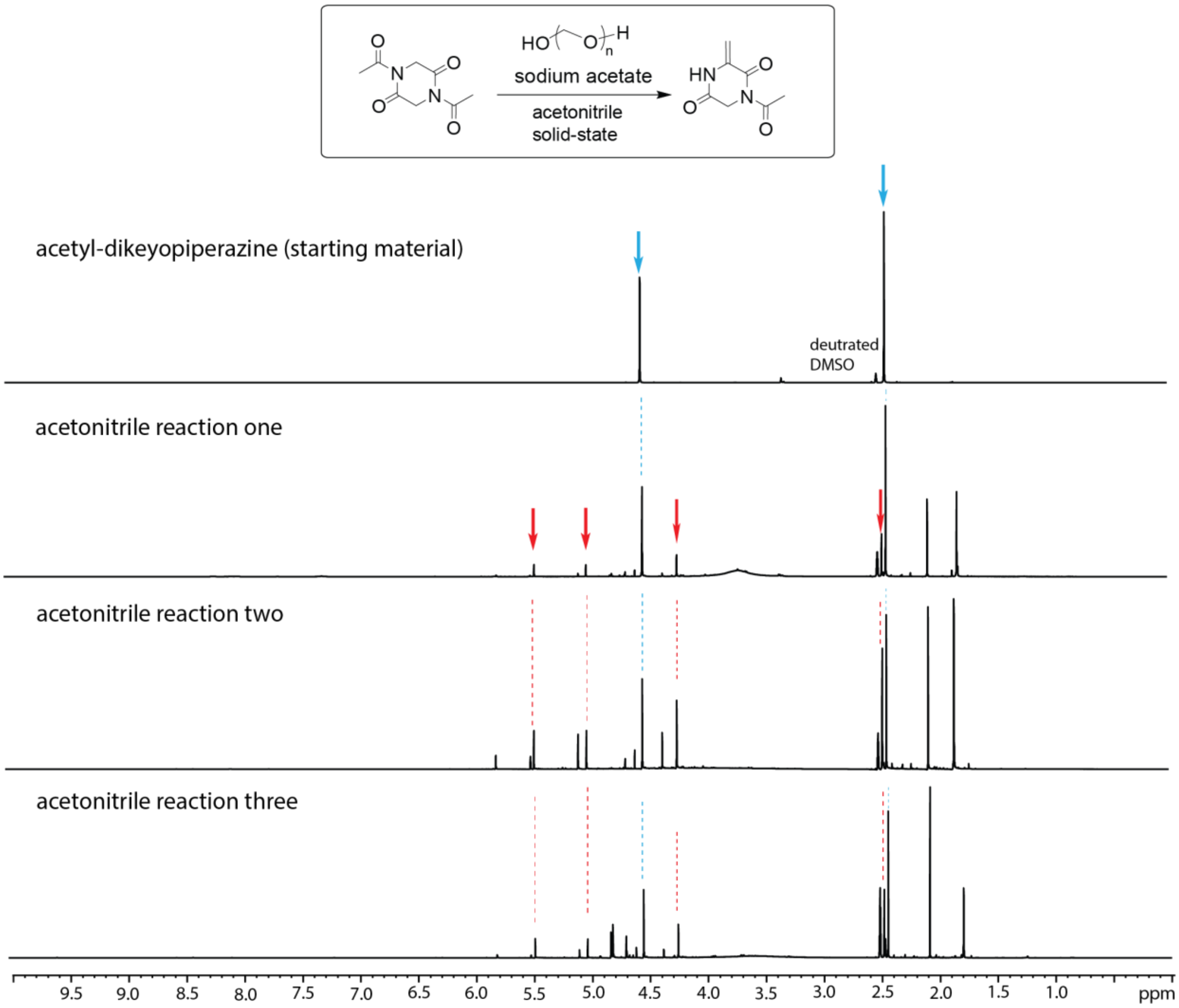

Figure S13 . ^{1}H NMR spectra (600 MHz, DMSO-d_6) of reaction mixtures after milling with acetonitrile as liquid additive. The starting material acetyl-diketopiperazine and cyclic N-acetylglycine dehydroalanine are labeled with blue and red arrows (as well as doted lines), respectively. Reaction conditions: N-acetyl cyclic glycine dimer (50 mg, 1 eq), paraformaldehyde (150 mg, ca. 20 eq), sodium acetate (150 mg), and acetonitrile (20 µL) at rt for 30 min in a 1.5 mL milling jar with stainless steel balls (one 3-mm and two 2-mm balls).

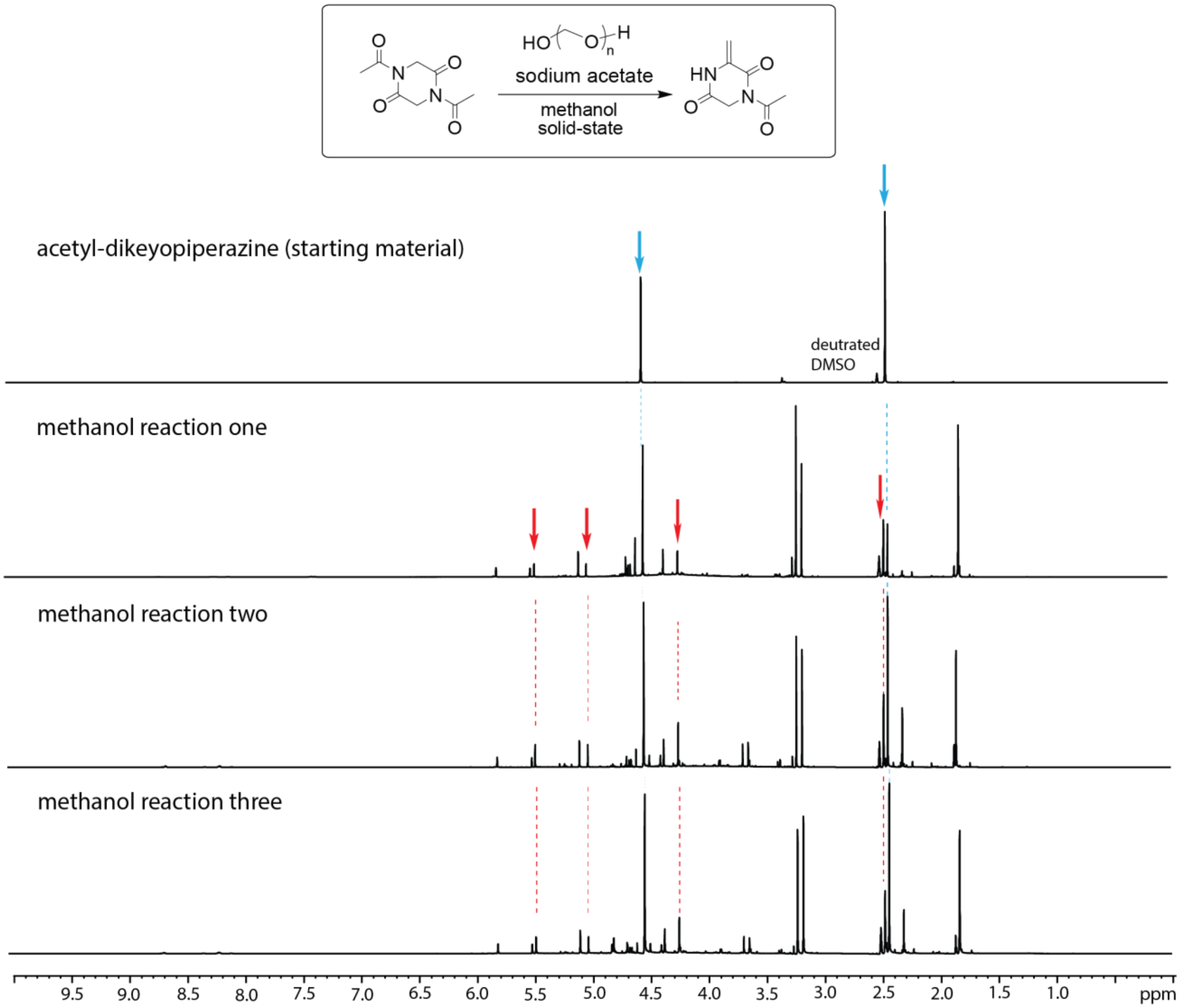

Figure S14 . ^{1}H NMR spectra (600 MHz, DMSO-d_6) of reaction mixtures after milling with methanol as liquid additive. The starting material acetyl-diketopiperazine and cyclic N-acetylglycine dehydroalanine are labeled with blue and red arrows (as well as dotted lines), respectively. Reaction conditions: N-acetyl cyclic glycine dimer (50 mg, 1 eq), paraformaldehyde (150 mg, ca. 20 eq), sodium acetate (150 mg), and methanol (20 µL) at rt for 30 min in a 1.5 mL milling jar with stainless steel balls (one 3-mm and two 2-mm balls).

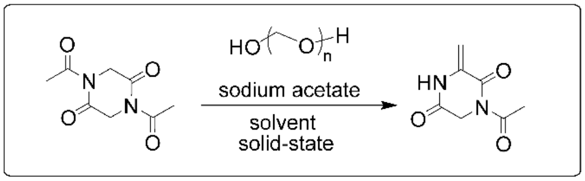

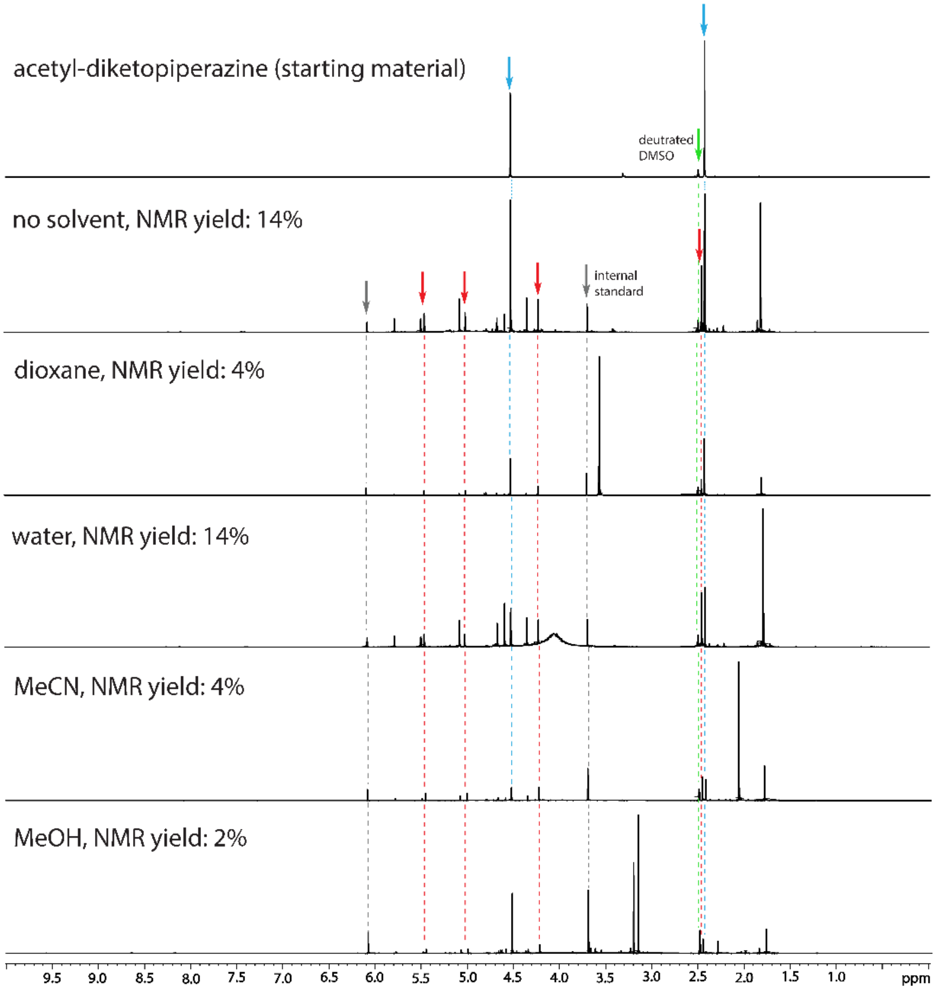

Figure S15. ^{1}H NMR spectra (700 MHz, DMSO-d_6) of reaction mixtures analyzed with 6.7 mM internal standard of 1,3,5-trimethoxybenzene in DMSO-d_6 after milling with various liquid additives. The starting material acetyl-diketopiperazine, cyclic N-acetylglycine dehydroalanine, and internal standard 1,3,5-trimethoxybenzene are labeled with blue, red, and grey arrows (as well as dotted lines), respectively. Reaction conditions: N-acetyl cyclic glycine dimer (50 mg, 1 eq), paraformaldehyde (150 mg, *ca*. 20 eq), sodium acetate (150 mg), and liquid additives (20 µL) at rt for 30 min in a 1.5 mL milling jar with stainless steel balls (one 3-mm and two 2-mm balls).

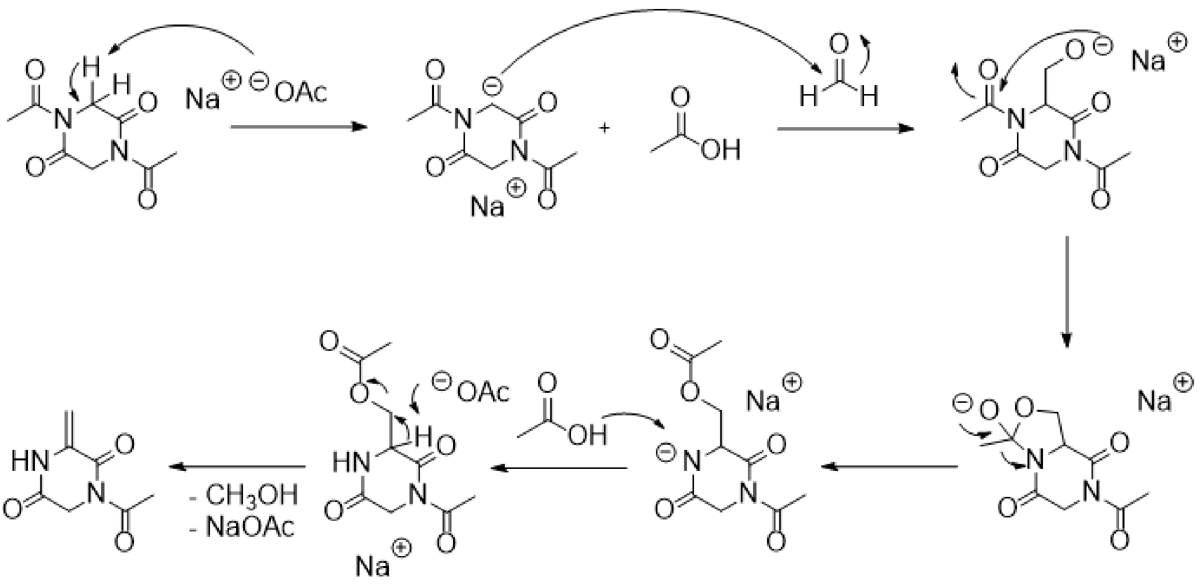

Figure S16. Proposed mechanism of *N*-acetyl cyclic glycine dimer undergoing condensation reaction with formaldehyde and NaOAc.

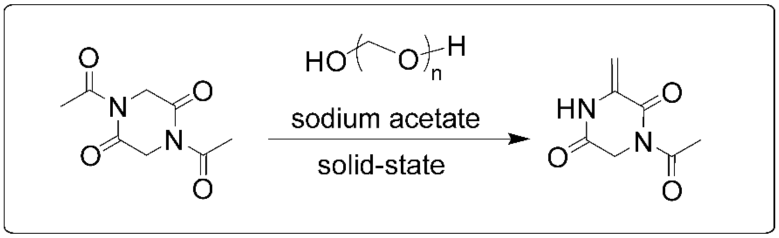

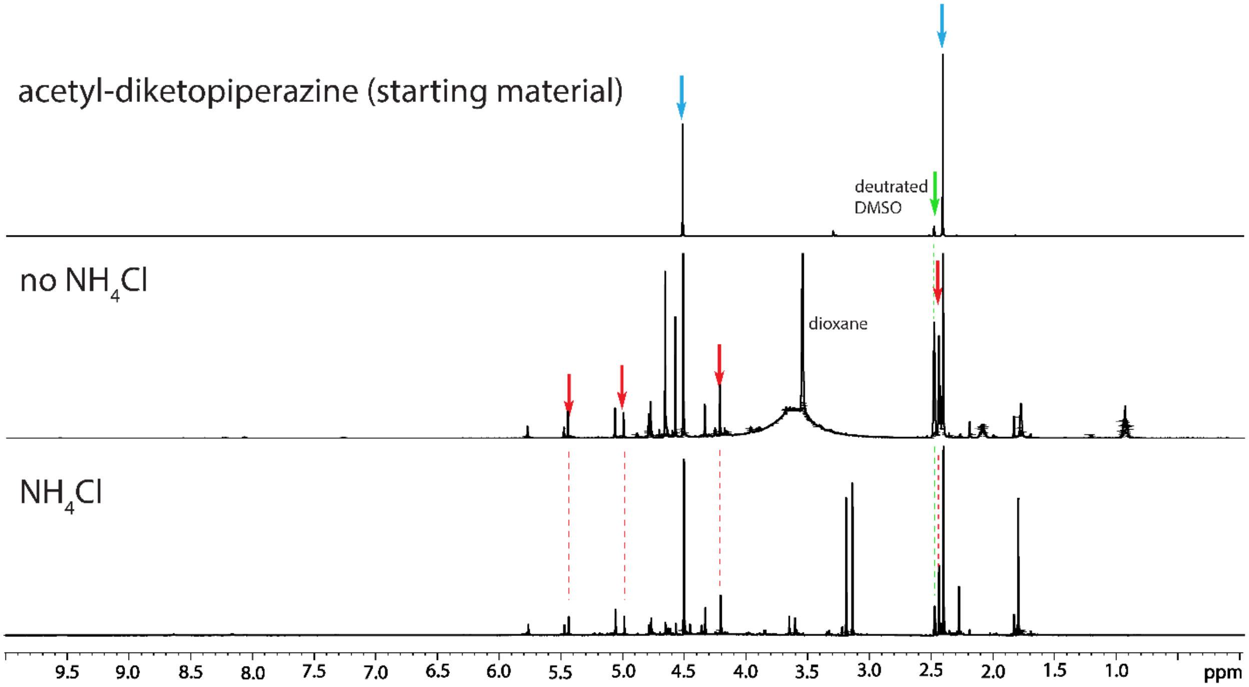

Figure S17.^{1}H NMR spectra (700 MHz, DMSO-d_6) of reaction mixtures analyzed with or without NH_4Cl after the milling process. The starting material acetyl-diketopiperazine and cyclic N-acetylglycine dehydroalanine are labeled with blue and red arrows (as well as dotted lines), respectively. Reaction conditions: N-acetyl cyclic glycine dimer (50 mg, 1 eq), paraformaldehyde (150 mg, ca. 20 eq), sodium acetate (150 mg, 7.3 eq), and dioxane (20 uL) at rt for 30 min in a 1.5 mL milling jar with stainless steel (one 3-mm and two 2-mm balls). After milling, NH_4Cl (535 mg, 40 eq) was added to the jars.

General Information

Materials and reagents

All chemicals were purchased from commercial suppliers unless otherwise noted, including paraformaldehyde, salts, and liquid additives. All chemical synthesis procedures were performed under air unless otherwise noted. *N*-acetyl cyclic glycine dimer (#A157857) were purchased from Ambeed.

Table S1. Summary of reaction screening (Figure 2–4).

entry	Figure #	conditions	conversions
1	2	NaOAc	100 ± 0
2	2	$Ca(OAc)_2$	41 ± 51
3	2	$Mg(OAc)_2$	34 ± 25
4	2	$In(OAc)_3$	0
5	2	$Bi(OAc)_3$	0
6	3	sodium propionate	83 ± 29
7	3	sodium lactate	0
8	3	sodium benzoate	27 ± 18
9	3	disodium oxalate	0
10	4	1,10-phenanthroline	0
11	4	*N*-methyl benzimidazole	0
12	4	proton sponge	0
13	4	phenazine	0

List of materials

Instrumentation

Ball milling

Mechanochemical reactions were performed on MM500 Vario (Retsch) and MM400 (Retsch). For reaction screening purposes, stainless steel balls (from Amazon vendors such as uxcell and QCQIANG) and a 1.5-mL milling metal jars (Retsch, #01.462.0230) were used. For large scale synthesis, 25-mL stainless steel jars (Retsch, #01.462.0213) with stainless steel balls was used instead of the 1.5-mL milling metal jars.

NMR

NMR was performed on Bruker AVANCE NEO 500, 600, and 700.

Experimental procedures

General procedure for solid-state reaction using ball mill

Details of equipment, accessories, and consumables are described in the instrumentation section. To a 1.5-mL metal jar, one 3.0-mm and two 2.0-mm balls were placed using tweezers. Powders of reactants such as *N*-acetyl cyclic glycine dimer (typically 50 mg) were placed in the jar using a weighing paper. Other reagents such as paraformaldehyde and various bases (typically 150 mg) were added in the jar using a weigh paper. The reaction was milled at 30 Hz without any temperature control. After the milling process, solvents such as deuterated solvents for NMR analysis was added, solid and liquid were separated using a countertop microcentrifuge, and the supernatant was used for analysis.

Solid-state reactions with liquid additive

N-acetyl cyclic glycine dimer (50 mg, 0.25 mmol), sodium propionate (150 mg, 1.56 mmol), and paraformaldehyde (150 mg, 5 mmol) were placed in 1.5 mL metal jar equipped with one 3.0-mm and two 2.0-mm balls. Liquid additive (20 µL) was added to the metal jar with a mechanical pipette. The reaction was milled at 30 Hz at room temperature. After the milling process, DMSO-d_6 was added, solid and liquid were separating using a countertop microcentrifuge, and the supernatant was analyzed by NMR.

Preparative synthesis of small molecules

Organic synthesis procedures

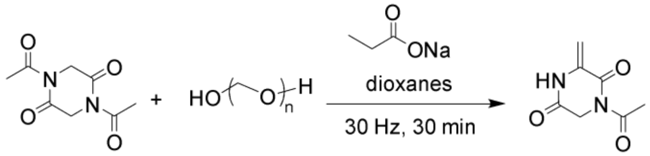

Preparative synthesis with sodium propionate: The procedure was optimized from the previous report.[1] *N*-acetyl cyclic glycine dimer (1.0 g, 5.04 mmol), solid paraformaldehyde (3 g, 100 mmol), and sodium propionate (3 g, 31.2 mmol) were placed in a 25 mL metal jar equipped with one 10.0-mm and two 8.0-mm stainless steel balls. Dioxanes (400 µL) was added to the metal jar with a mechanical pipette. The mixtures were milled for 30 minutes at 30 Hz at room temperature. The reaction mixture was suspended in DMF (200 mL) and the supernatant was decanted with a paper filter to separate the solid. The addition of silica gel (*ca.* 3 mL) to the supernatant, the DMF mixture was dried under vacuum. The resulting mixture was purified through silica gel column chromatography (2:1 hexane/ethyl acetate). Crystalline solids obtained during solvent removal under vacuum after silica gel column chromatography was then triturated with water to remove residual impurities to afford the product as a white solid (280 mg, 33%). Spectral data in DMSO-d_6 is consistent with a previous report.[1] ^{1}H NMR (700 MHz, DMSO-d_6): δ 10.78 (br, 1H), 5.46 (s, 1H), 5.01 (s, 1H), 4.24 (s, 2H), 2.47 (s, 3H).

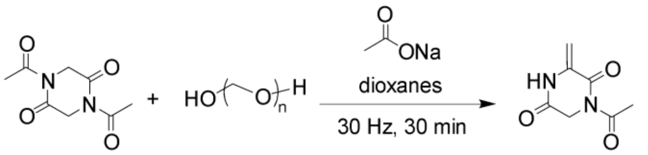

Preparative synthesis with sodium acetate: The procedure was optimized from the previous report.[1] *N*-acetyl cyclic glycine dimer (1.0 g, 5.04 mmol), solid paraformaldehyde (3 g, 100 mmol), and sodium acetate (3 g, 37.0 mmol) were placed in a 25 mL metal jar equipped with one 10.0-mm and two 8.0-mm stainless steel balls. Dioxanes (400 µL) was added to the metal jar with a mechanical pipette. The mixtures were milled for 30 minutes at 30 Hz at room temperature. The reaction mixture was suspended in DMF (200 mL) and the supernatant was decanted with a paper filter to separate the solid. The addition of silica gel (*ca.* 3 mL) to the supernatant, the DMF mixture was dried under vacuum. The resulting mixture was purified through silica gel column chromatography (2:1 hexane/ethyl acetate). Crystalline solids obtained during solvent removal under vacuum after silica gel column chromatography was then triturated with water to remove residual impurities to afford the product as a white solid (237 mg, 28%). Spectral data in DMSO-d_6 is consistent with a previous report.[1] ^{1}H NMR (700 MHz, DMSO-d_6): δ 10.78 (br, 1H), 5.46 (s, 1H), 5.01 (s, 1H), 4.24 (s, 2H), 2.47 (s, 3H).

NMR spectra of synthesized compounds

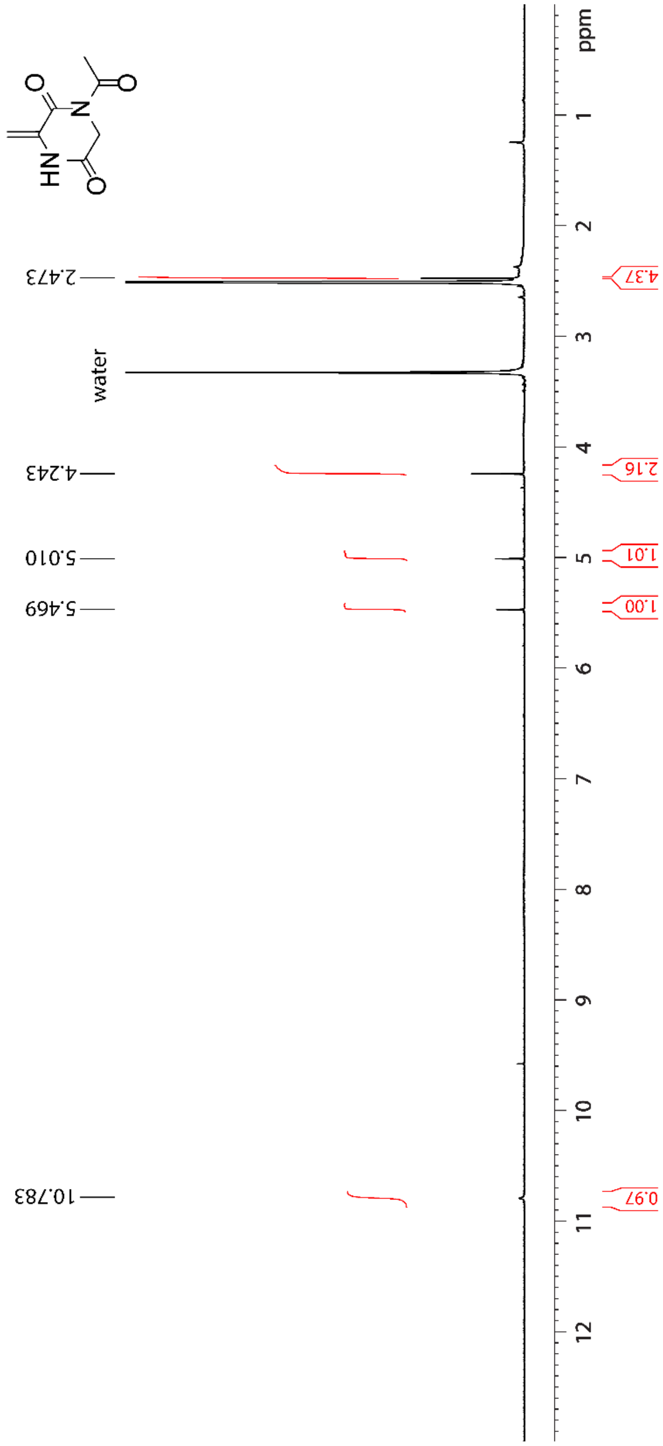

Figure S18 . ^{1}H NMR spectrum (700 MHz) of cyclic N-acetyl glycine dehydroalanine in DMSO-d_6

References for Supporting Information

(1) Thuan, J. C.; Koirala, A.; Pirez, J.; Ohata, J. Prebiotic Roles of Formaldehyde as an Activating Agent and a Building Block in Solid-State Peptide Modification. *ACS Bio Med Chem Au* **2025**, *5* (6), 982–993. https://doi.org/10.1021/acsbiomedchemau.5c00167.
